# Less is better than more with resection of periacetabular tumors – A retrospective 16 years study and literature review

**DOI:** 10.3389/fsurg.2022.1036640

**Published:** 2022-12-08

**Authors:** Ran Atzmon, Michael Drexler, Oleg Dolkart, Yariv Goldstein, Jeremy Dubin, Amir Sternheim, Yair Gortzak, Jacob Bickels

**Affiliations:** ^1^ Orthopedic Department, Assuta Ashdod Medical Center, Ashdod, Israel, affiliated to Beer Sheva Faculty of Medicine, Beer Sheva University, Israel; ^2^ Division of Orthopedic Surgery, Tel Aviv Sourasky Medical Center, affiliated to the Sackler Faculty of Medicine, Tel Aviv University, Tel Aviv, Israel; ^3^ The National Unit of Orthopedic Oncology, Tel Aviv Sourasky Medical Center, affiliated to the Sackler Faculty of Medicine, Tel Aviv University, Tel Aviv, Israel; ^4^ Hillel-Yaffe Medical Center, Orthopedic Oncology Unit, Department of Orthopaedic Surgery, Affiliated with the Technion - Israel Institute of Technology, Hadera, Israel

**Keywords:** resection, reconstruction, periacetabular tumors, bone tumor - osteosarcoma, hip joint, hip

## Abstract

**Introduction:**

Wide resections of periacetabular tumors create a sizeable bony defect that inevitably results in severe loss of function. Reconstruction of such defects usually requires using large metal implants, a feature associated with considerable surgery extension and complications. The aim of this study is to report resection with no reconstruction of the bony defect. In this retrospective study, we reviewed a consecutive series of 16 patients diagnosed with malignant periacetabular tumors and underwent en-bloc resection without reconstructing their remaining bone defect.

**Methods:**

Records were reviewed of 16 consecutive patients diagnosed with malignant periacetabular tumors and underwent en-bloc resection without reconstructing their remaining bony defect. Measurements included: the duration of surgery, blood loss, hemoglobin levels and the need for blood transfusions, data on other hospitalization characteristics, and intraoperative and postoperative complications.

**Results:**

Sixteen patients with malignant periacetabular bone tumors and extensive bone destruction underwent wide periacetabular tumor resection with a mean follow-up of 75 months and a mean age of 53 years. The average HOOS score was 46 (range: 20 to 76), and the mean MSTS score was 13% (range: 0 to 15). The mean operative time was 4.1 h, and the mean blood loss was 1200 ml. At their most recent follow-up, patients had a mean shortening of their operated extremity of 4.8 cm, and all could ambulate with assisting devices.

**Conclusion:**

Wide resection of periacetabular tumors without reconstruction provides acceptable levels of function and was associated with shorter surgical time, less blood loss and fewer postoperative complications compared to resection with reconstruction. Therefore, this approach may be considered a viable surgical option in patients with an extensive malignant periacetabular.

**Level III:**

Retrospective study.

## Introduction

Wide resections of malignant periacetabular tumors, which are usually associated with extensive bone destruction and soft-tissue extension, necessitate en-bloc resection of the acetabulum and the enveloping muscle and ligaments. This procedure results in a loss of the hip joint and mechanical dissociation between the pelvic girdle and the lower extremity. These periacetabular resections may be performed as a single-stage or two-stage procedure in which reconstruction is done in a later stage. A single-stage resection of a periacetabular tumor usually utilizes endoprostheses or allografts for reconstruction. Thus requires a considerable extension of the surgical time and is associated with a prolonged period of rehabilitation and considerable complications, including; infection, delayed wound healing, prosthetic dislocation and loosening, and impaired functional outcome (1–6). Therefore, we hypothesized that performing wide resection of these tumors without reconstruction would result in a shorter operation, less intraoperative blood loss, fewer postoperative complications, and an acceptable level of function. This study describes the clinical and functional outcomes of a series of patients who underwent resection of a malignant periacetabular tumor without defect reconstruction.

## Patients and methods

Medical records of 16 patients who underwent resection of a periacetabular tumor were retrospectively evaluated for the operative reports, postoperative recovery and rehabilitation reports, and functional outcome. The study was conducted in a single center from 1998 to 2014 by fellowship-trained orthopedic oncologists and was approved by the institutional review board. The type of resection was classified according to Enneking's and Dunham's classification system (4). Functional outcomes were assessed at the last pre-operative visit and after the surgery at 6 and 12 months, using the Hip Disability and Osteoarthritis Outcome Score (HOOS) (7). And the Musculoskeletal Tumor Society Scoring (MSTS) system, which is a clinician-scored system for assessing pain, function, and emotional acceptance on the part of the patients (8). Postoperative Leg length discrepancy (LLD) was also measured.

Demographic information, such as age, gender, and comorbidities, were taken from the Charlson Comorbidity Index (CCI) (9), along with operational data recorded by the surgical staff and physical status graded by the American Society of Anesthesiologists (ASA) physical status classification system (10, 11). In addition, the surgical duration, blood loss, hemoglobin levels and the need for blood transfusions, data on other hospitalization characteristics, and intraoperative and postoperative complications were retrieved from the medical files.

The inclusion criteria were the following: i) patients who could withstand surgery according to the pre-operative evaluation by the surgeon and anesthesiologist, ii) utilization of all other conservative treatments available, including chemotherapy and radiation, iii) patients who gave their consent to a salvage procedure after all the alternatives been explained to them by the operating staff, iv) life expectancy of at least two years and v) a minimal Enneking score of 2. The exclusion criteria included: i) iliac resection at the sacroiliac joint and reconstruction with a saddle prosthesis, and ii) life expectancy below two years.

Prior to the surgery, all the patients underwent a comprehensive clinical, radiological and laboratory evaluation to evaluate the extent of the tumor, its neurovascular involvement, and other comorbidities. They were followed by a multidisciplinary team, including the operating orthopedic oncologist surgeon, radiologist, oncologist, and social worker, who decided on the appropriate adjuvant treatment therapy (i.e., chemotherapy regimen, social support, etc.'). In cases when main arteries or nerves were affected, Vascular surgeons and peripheral nerve surgeons specialists were also involved.

### Surgical technique

**Figure 1:**
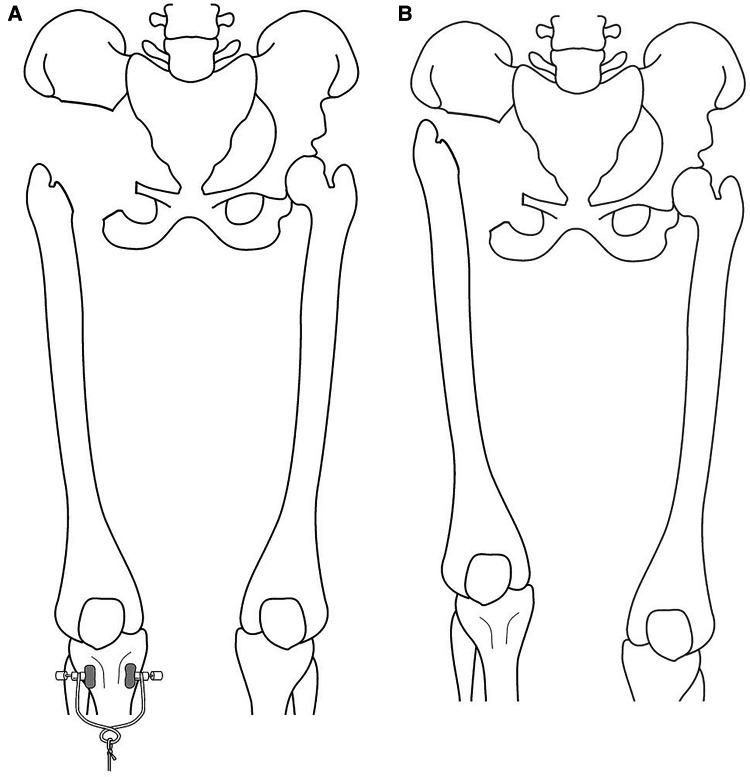
**(A**) illustration of the surgical procedure under skeletal traction, showing the remaining bone defect following resection. (**B**) Illustration of the surgical procedure after the bony resection and traction removal. The traction allows the bone defect to be filled with reactive tissue, creating a mass effect to reduce the upward migration and decrease the LLD. *LLD - Leg length discrepancy.

Patients were positioned in the lateral decubitus position with a posterior tilt to maximize anterior exposure. A utilitarian pelvic incision was used to expose both the anterior (internal) and posterior (extrapelvic) aspects of the pelvis. An ilioinguinal incision was additionally used to develop the retroperitoneal plane, and a posterior gluteus maximus fasciocutaneous flap was used to develop the retrogluteal space. Total hip exposure was used to identify the sciatic nerve and the posterior acetabular column. A pad was placed within the sciatic notch to protect the sciatic nerve and superior gluteal vessels. Three osteotomies were required and utilized for periacetabular resection: supra-acetabular, superior pubic ramus, and ischial osteotomy. The iliac vessels were identified, marked, and mobilized, and the hypogastric artery was identified and ligated. Further blood control was achieved using electrocauterization and packing as needed. The osteotomy level through the ilium was determined from within the pelvis, as was the superior pubic ramus osteotomy. The external rotator muscles were dissected, and the hip joint capsule was opened and the femoral head was dislocated. The anterior and posterior acetabular columns were exposed to allow osteotomy of the acetabulum. Intra- or extra-articular resections of the hip joint were done based on the presence of the tumor within the hip joint. Following tumor resection, skeletal traction was applied to the operated extremity for three weeks to allow formation of tissue scarring around the pelvic girdle's bony defect, creating a mass effect to minimize proximal migration of the operated extremity and subsequent leg length discrepancy. This was followed by traction removal and gradual full weight-bearing. (Figures 1A,B). After the surgery, the patients remained in the hospital for 3 weeks until the skeletal traction was removed. Additional discharge criteria were adequate general and wound healing process, stable vital signs and blood tests, proper nutrition, and no major complication.

### Postoperative evaluation

Patients were evaluated at 2 weeks, 6 weeks, 3 months, 6 months, 9 months and one year postoperatively (range:12 to 192 months; mean 75 months). Physical examination, plain radiography, and computerized chest tomography were performed at each visit. In addition, the orthopedic oncologist and oncologist evaluated the patients semiannually for an additional three years and annually thereafter, analyzing the clinical records, operative reports, and imaging studies.

### Limb salvage failure

The definition of an unsuccessful limb salvage surgery was determined by the need for amputation for any reason, i.e.,; Time To Event (TTE). The Kaplan-Meier product-limit method was used to determine patient and limb survival rates (10). All patients were evaluated based on their most recent clinic visit status. The data on patients who passed away due to the original malignancy without having undergone amputation was retrieved from their last clinic visit.

### Statistical analysis

The chi-squared or Fisher exact test was applied for categorical variables, and Student's *t*-test or ANOVA was used for scale variables at a significance level of *p* < 0.05. IBM SPSS Statistics, version 21 for Windows (SPSS, Chicago, IL) was used for all analyses. In addition, a Kaplan-Meier survivorship analysis was performed with calculated 95% confidence intervals (CI) for the amputation for any cause as an endpoint.

## Results

**Figure 2:**
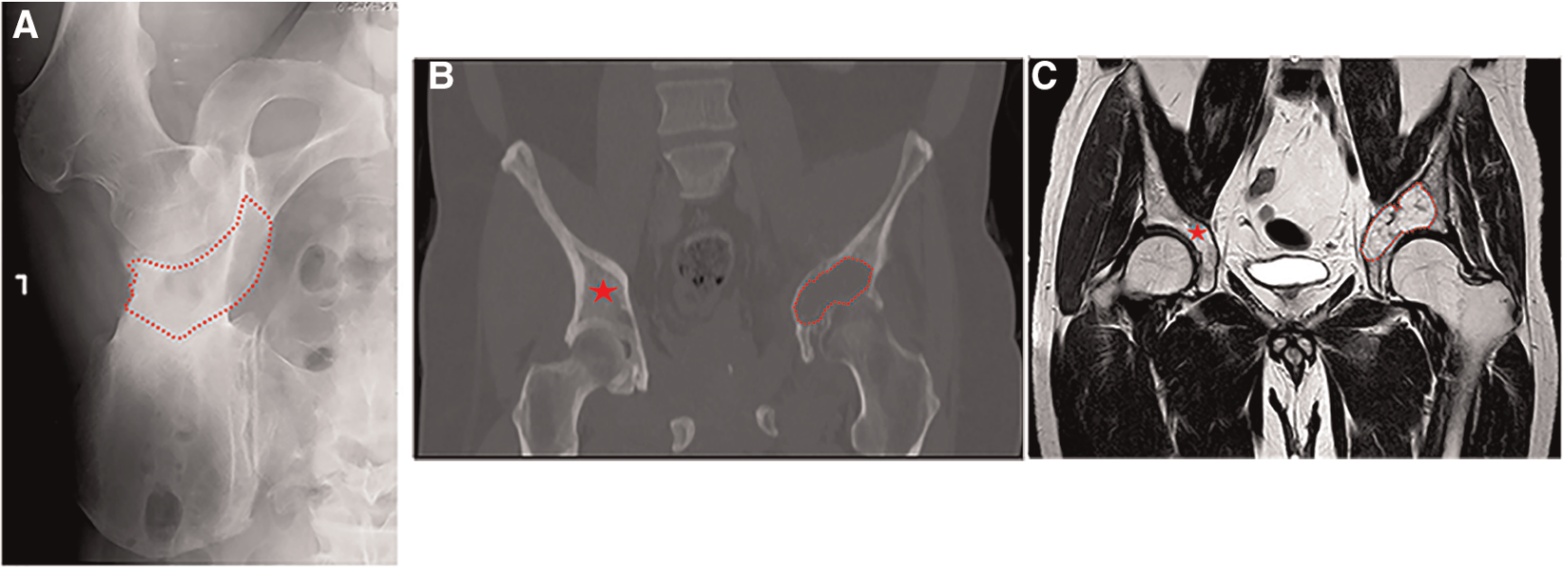
(**A**) plain anterior-posterior (AP) radiograph of a left hemipelvis, showing high-grade chondrosarcoma of the acetabulum with the destruction of the medial acetabular wall. The red dotted line delimits the radiolucent area created by the bone tumor. (**B,C**) A coronal computed tomography (CT) and T1 sequence of magnetic resonance imaging (MRI) retrospecivaly, of high-grade chondrosarcoma of the acetabulum with destruction of the medial acetabular wall (red dotted line) and the contralateral healthy bone area (red asterisk).

Sixteen patients with malignant periacetabular bone tumor who presented with extensive bone destruction and tumor extension into the surrounding soft tissues, underwent wide periacetabular tumor resection (Figures 2A,B). The cohort comprised of 10 males and 6 females with a mean age of 53 years (range: 22–83). The histological diagnoses are summarized in Table 1.

**Table 1:** Diagnosis on admission and tumor classification based on grade and stage.

Age (years)/gender	Diagnosis	Resection
56/M	Ewing's sarcoma	I,II
64/M	Chondrosarcoma	I,II,III
78/F	Chondrosarcoma	I,II,III,IV
73/M	Metastatic RCC	I,II,III
65/M	Chondrosarcoma	I,II,IV
24/M	Osteosarcoma	I,II,III,IV
74/M	Osteosarcoma	I,IV
83/F	Osteosarcoma	I,II,III,IV
28/F	Ewing's sarcoma	I,II,III
28/M	Osteosarcoma	I,II
50/F	Osteosarcoma	I,II,IV
60/M	Ewing's sarcoma	II,III
59/M	Chondrosarcoma	II,III
47/M	Chondrosarcoma	I,II,IV
57/M	Osteosarcoma	II,III
22/F	Osteosarcoma	I,II,IV

M, male; F, female; RCC, renal cell carcinoma.

Demographic information, including age, gender, comorbidities by CCI, and the ASA physical status are summarized in Table 2. The average functional data documentation was a mean of 75 months (range: 12 to 192 months). The average HOOS score was 46 (range: 20 to 76). The mean MSTS score was 13% (range: 0 to 15), and function was considered fair in four patients (33.3%) and poor in eight (66.5%). Surgical information is summarized in Table 3. The mean operative time was 4.1 h (range: 2 to 7.5), and the mean estimated volume of blood loss during surgery was 1200 ml (range: 500 to 2750). At the time of the study compilation and review of the findings, 10 patients (62%) had passed away of their disease, of which 6 succumbed to osteosarcoma, 3 to chondrosarcoma and 1 to Ewing's sarcoma. Two of the 16 patients (12.5%) had a local recurrence and underwent amputation at 64 and 144 months following the index surgery. The remaining 14 patients (87.5%) maintained a viable limb, yielding an 87.5% limb survival rate at 16 years.

**Table 2:** Patient demographics.

		Total
Gender, *n* (%)		
	Female	6 (37.5)
	Male	10 (62.5)
Side		
	Right	9 (56)
	Left	7 (44)
ASA, (average)		2–3 (2.4)
	0	2 (12.5)
	1–3	9 (56.25)
	4+	5 (31.25)
CCI score*, range (average)		2–6 (3.4)

*CCI score - Charlson Comorbidity Index.

**Table 3:** Surgical results.

	Total
Operation length, hours, average (range)	4.1 (2 to 7.5)
Blood loss, ml, average (range)	1200 ml (500 to 2750)
Intra-operative packed blood cells, units (range)	11 (3 to 6)
Non-orthopedic complications, n*	6 (37%)
Hospitalization days, average (range)	34.5 (20 to 88)

*Pneumonia, urinary tract infection, myocardial infarction, pulmonary edema, deep vein thrombosis and pulmonary emboli.

### Complications

Two patients suffered from intraoperative injury to the urinary bladder which required an acute repair and postoperative continuous urinary catheterization for an additional 6 weeks. The mean pre and post-hospitalization period for these patients was 34.5 days (20 days to 88 days). Three patients had a deep infection at the operative site, and two of them required a repeat surgical debridement procedure. One patient suffered from a deep vein thrombosis (DVT), diagnosed with Ultra-Sound and treated with Enoxaparin sodium.

**Figure 3:**
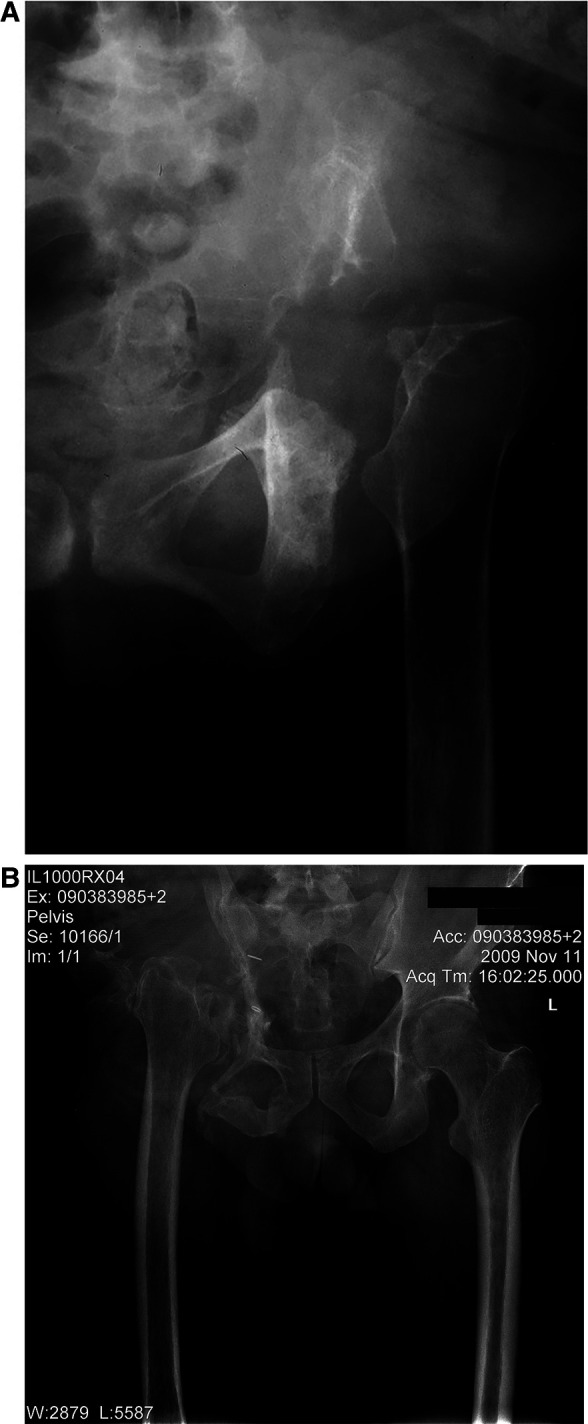
(**A,B**) plain anterior-posterior (AP) radiograph of the hemipelvis showing proximal migration of the operated extremity after bone tumor resection following six weeks of weight bearing. (**A**) shows the left hemipelvis, and (**B**) the right hemipelvis.

**Figure 4:**
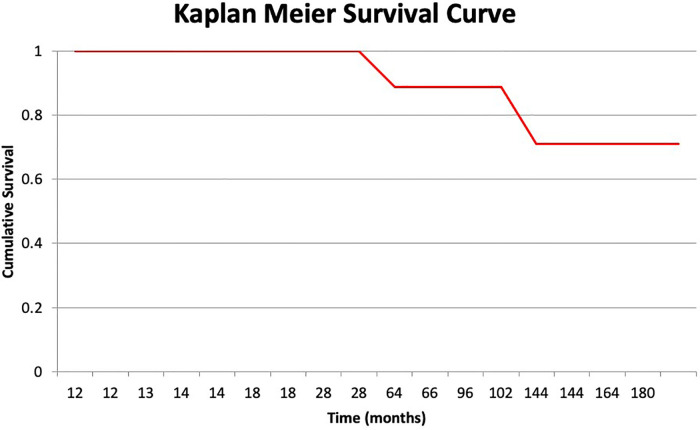
Kaplan-Meier survivorship curve of study patients.

At their most recent follow-up, all the patients were instructed to use shoe lifts, and all could ambulate using assistive devices (either a cane or crutches), except for two patients who required a walker. One of the latter two occasionally used a wheelchair for prolonged walking. The mean shortening of the operated extremity was 4.8 cm (range: 4 cm to 6 cm) (Figure 3A,B). The survival rate for the limb with amputation as the endpoint was 88.9% (Figure 4).

## Discussion

Resections of periacetabular tumors with an endoprosthetic reconstruction remain the standard of care for most patients with a primary bone sarcoma involving the acetabular area. Unfortunately, these procedures are often fraught with high rates of perioperative complications, including considerable blood loss and extended operative time, high rates of postoperative complications, poor outcomes, and long-term failure (12–16) (Table 4).

**Table 4:** Surgical details and complications.

Study	Patients (*n*)	Type of Reconstruction	Surgical Time hrs (range)	Blood loss ml (range)	Follow-up Months (range)	Complications *n* (%)	MSTS*
Bus et al. (12)	19	Prosthesis	N/A	N/A	39 (1 to 103)	15 (79)	49%
Delloye et al. (17)	24	Allograft	10 (4.5 to 16.5)	4,359 (1,000 to 11,300)	41 (1 to 137)	12 (50)	73%
M Rudert et al. (18)	38	Prosthesis	N/A	N/A	N/A	N/A	43.7%
Dasen Li et al. (19)	17	Prosthesis	N/A	N/A	35 (10–75 months)	N/A	63.5% ± 10.8%
Tomohiro Fujiwara et al. (20)	21	Prosthesis	N/A	v	52 (6–233)	8 (40)	66%
	54				79 (6–256)	24 (44)	65%
Victor Housset et al. (21)	33	Endoprostheses & Allograft–prosthesis	5 (3.8–6)	3–5 PRBC** transfusion	76 (24–220)	< 16 (50)	N/A
Donati et al. (13)	35	Prosthesis-allograft composite	6 (5 to 7)	N/A	121 (61 to 188)	21 (60)	72%
Jansen et al. (14)	17	Prosthesis	6.3 (6 to 11)	N/A	94 (2 to 204)	14 (82)	47%
Wang et al. (15)	50	Prosthesis	6.8 (4 to 13)	4,200 (600 to 20,000)	54 (12 to 113)	33 (66)	61.4%
Tomohiro Fujiwara et al. (22)	65	Custom-made prosthesis	N/A	N/A	85 (4 to 435)	40 (62)	59%,
	21	Prosthesis	N/A	N/A		5 (24)	74%
	18	Autograft	N/A	N/A		10 (56)	64%
	18	No reconstruction	N/A	N/A		3 (17)	72%
Witte et al. (2)	40	Prosthesis			24 (1 to 61)	30 (75)	50%
Current study	16	No reconstruction	4.1 (2 to 7.5)	1,200 (500 to 2750)	75 (12 to 192)	6 (37)	13%

*MSTS, musculoskeletal tumor society scoring system.

**PRBC, packed red blood cells.

Moreover, many patients require additional surgical procedures, including complete leg amputation, which prevents the patient from achieving satisfactory well-being post-surgery (23, 24). However, the literature does not contain comprehensive data regarding surgical resection of periacetabular tumors without reconstruction.

Complications after a periacetabular tumor resection are wildly described throughout the literature. Complications rates in patients who undergo periacetabular tumor resection are as high as 82% for some reconstructive procedures (12–22, 24–30) (Table 4). These complications may gravely impact the overall results because they present further compromise of an already impaired quality of life and may delay the initiation of adjuvant oncologic treatment (e.g., chemotherapy and radiation). Witte et al. (16). reported a 75% complication rate in 40 patients who underwent reconstruction procedures using a hemipelvis endoprosthesis. Wang et al. (15) reported a 66% complication rate in 50 patients treated with a modular hemipelvis endoprosthesis. Jansen et al. (14) reported a complication rate of 82% in 17 patients treated with a saddle prosthesis. Furthermore, Delloye et al. (17) reported a complication rate of 50% in 24 patients who underwent reconstruction using a massive bone allograft. A recent study found pelvic prosthesis for reconstruction after resectioning a pelvic tumor using a 3D-printed pelvic endoprosthesis was safe, without additional complications, and provided good short-term functional results (31). However, the novelty of 3D-printing techniques limits most comparisons in the literature. Infection is one of the most prevalent complications, which tremendously affects the patients' well-being, hospitalization time, and revision surgery (20–22, 32). Ogura and colleagues (32). conducted a nationwide survey of prosthetic reconstruction performed on 80 patients following resection of periacetabular tumors in 17 institutions. The authors reported on deep infection rate of 39% with a mean follow-up period of 65 months. Housset et al. (21) reported on 33 patients with primary malignant bone tumors involving the proximal femur or acetabular who underwent resection and reconstruction, with a mean follow-up of 76 months. The total infection rate was 27.3%, with 21.2% of deep infection. In a recent study by Fujiwara et al. (20) the authors reported on 75 patients who underwent resection and reconstruction due to bone sarcoma, with a mean follow-up period of 73 months (range, 6– 256 months). The most common complications were dislocation and deep infection, accounting for 28%. Another study by Fujiwara and colleagues, which included 122 patients with a mean follow-up was 85 months (4 months to 36.3 years), compared different reconstruction methods of 104 patients and nonskeletal reconstruction of 18 patients, following resection. In this study, deep infection was the most common complication with a 26% incidence. Interestingly, deep infection did not occur in patients with nonskeletal reconstruction (22). In the current study 3 patients (19%) had a deep infection, this might be attributed to the versatility of the different types of sarcomas included in the study and the substantial insult to the surrounding soft tissue. Nonetheless, these numbers are significantly low compared to the reported literature on reconstruction surgeries (20–22, 32). In contrast, the reported postoperative complication rates for resectioning periacetabular tumors without reconstruction are much lower. Carmody et al. (26) reported complications in only 2 of their 5 patients (40%), Griesser et al. (27) reported a complication rate of 33% in 4 out of 12 patients, while Ogura et al. (32) found a complication rate of 59% in the 80 patients studied.

In our study, the complication rate was found to be 37.5% in 6 out of 16 patients, This finding demonstrates favorable outcomes using periacetabular tumor resection without reconstruction, compared to the aforementioned published data, and giving these patients a small window to live comfortably after their surgery.

In addition to complications, surgical time in the operation room and blood loss are outcomes that favor resections without reconstruction. Patients who undergo periacetabular tumor resection with reconstruction have longer surgical time and higher blood loss than those whose operation does not include reconstruction (12–17). In a study by Bell et al. (2) seventeen patients with a high-grade sarcoma of the pelvis were operated, of whom nine were reconstructed with an allograft. The mean operative time for these patients was 9.1 h (range: 7 h to 11.5 h), and the mean blood loss was 4700 ml (range: 1500 ml to 11000 ml). Kitagawa et al. (6) also reported a relatively prolonged operative time of 6.5 h (range: 5 h to 8.18 h), and heavy blood loss of 13 units of blood (range 5 units to 26 units) in their series of 16 patients who underwent resection of periacetabular tumors and reconstruction with a saddle prosthesis.

Likewise, a similar prolonged operative time and blood loss were reported by Delloye et al. (17). who used massive bone allografts for reconstruction in their reported series of 24 patients. Their mean operative time was 10 h (range: 4.5 h to 16.5 h) and the mean blood loss was 4359 ml (range: 1000 ml to 11300 ml). In a study by Wang et al. (15) fifty patients were reconstructed with a modular tumor prosthesis. The mean operative time and blood loss were 6.8 h (range: 4 h to 13 h) and 4200 ml (range: 600 ml to 2000ml), respectively. In this study, the mean operative time was found to be 4.1 h (range: 2 h to 7.5 h), and the mean blood loss was 1200 ml (range: 500 ml to 2750 ml). Our results support previously published data by Hu et al. (30) who operated on 27 patients who underwent resection without reconstruction. The mean operative time was 2.8 h (range: 2 h to 5.8 h), and the mean blood loss was 1200 ml (range: 600 ml to 2200 ml).

On the other hand, periacetabular tumor resection without reconstruction did show similar LLD but different MSTS compared to the literature (19, 30). In our study, the mean LLD was 4.8 cm (range 4 cm to 6 cm), and the MSTS score was 13%. Previously published studies with resection of periacetabular tumors without reconstruction have shown similar LLD results, but significantly higher postoperative MSTS score, Hu et al. (30) reported a mean LLD of 5 cm (range 2 cm to 7.5 cm) and an MSTS score of 75.6%. Griesser et al. (27) reported a mean MSTS score of 45%, and Carmody et al. (26) reported a mean MSTS score of 43.3% in their non-reconstruction groups. Notably, prosthetic reconstruction of the periacetabular area may also be associated with fewer extremity length discrepancies; Aljassir et al. (1) reported a mean limb shortening of 3 cm (range 1 cm to 6 cm) of the operated leg and a mean MSTS score of 50.8% in their series of 27 patients who were reconstructed with a saddle prosthesis after periacetabular tumor resection. Bus et al. (12) reported a mean MSTS score of 49% in 19 patients who underwent periacetabular reconstruction with a pedestal cup endoprosthetic. And Donati et al. (13) reported a mean MSTS score of 72% among 35 patients who underwent periacetabular tumor resection reconstruction with an allograft prosthetic composite. However, we maintain the importance of reduced hospitalization time and fewer postoperative complications in this salvage surgery.

### Study limitations

We acknowledge some limitations in the study. The patient population was relatively small and heterogeneous, making it challenging to establish statistical significance and deduce firm conclusions. However, our study is focused on the surgical technique and its associated postoperative complications and function, parameters on which the histological type of the malignant tumor treated has limited impact. Moreover, the decision not to reconstruct does not affect the oncological outcome (i.e., local tumor recurrence and metastatic dissemination). In addition, we did not include a control group, which is of interest in future studies, especially regarding patients' postoperative quality of life. However, we were able to remark on a procedure that has a limited scope in the literature and scantly describes it. The tumor's type and location require that each surgery be tailormade to the patient's condition and functional demand, making it difficult to standardize the surgical resection procedure. In addition, adjuvant treatments, which contribute to complication rates and functional outcomes, vary greatly between patients, depending on tumor grade and stage and the patient's general condition. Unfortunately, many patients do not overcome their disease, thus making long-term follow-up of functional analysis difficult to obtain. We acknowledge the disparate MSTS score compared to previous studies but can value the shorter operation time, less blood loss, and fewer postoperative complications in a salvage surgery situation. Given the low life expectancy of patients with malignant periacetabular tumors, we believe the net benefit of the complete resection without reconstruction outweighs the lower satisfaction score and acts as a viable option overall.

## Conclusion

This study demonstrated that resection of periacetabular tumors without reconstruction is associated with shorter operation time, less blood loss, and fewer postoperative complications, leading to earlier discharge to the patient's homes and an acceptable level of function when compared to a single-stage surgery in which reconstruction is performed. The major drawback of this approach is a limb length discrepancy resulting in a lower satisfaction rate. Therefore, resection without reconstruction of periacetabular tumors should be considered in patients who are at high risk for extensive surgery.

## Data Availability

The raw data supporting the conclusions of this article will be made available by the authors, without undue reservation.
